# Effect of Fullerenol C_60_(OH)_24_ on Viability and Phagocytic Activity of Human Neutrophils

**DOI:** 10.3390/nano16070405

**Published:** 2026-03-27

**Authors:** Sergey Lazarev, Valeria Timganova, Maria Bochkova, Maria Dolgikh, Darya Usanina, Svetlana Zamorina, Mikhail Rayev

**Affiliations:** 1 Institute of Ecology and Genetics of Microorganisms, Perm Federal Research Center of the Ural Branch of the Russian Academy of Sciences, 614081 Perm, Russia; lasest1999@gmail.com (S.L.); timganovavp@gmail.com (V.T.); krasnykh-m@mail.ru (M.B.); dolgikhtatanya@gmail.com (M.D.); usanina_d@mail.ru (D.U.); zamorina.sa@gmail.com (S.Z.); 2 Department of Biology, Perm State University, 614086 Perm, Russia

**Keywords:** fullerenol, human neutrophils, cell viability, phagocytic activity, reactive oxygen species, antioxidant activity

## Abstract

Water-soluble fullerene derivatives such as fullerenol C_60_(OH)_24_ are promising candidates for nanomedicine applications, yet their effects on innate immune cells remain poorly characterized. We investigated the interaction of fullerenol with human neutrophils isolated from healthy donors, exposed to concentrations of 0.25–200 μg/mL over 24–72 h. Using multi-parameter flow cytometry, we assessed viability, apoptosis, phagocytic activity, and intracellular reactive oxygen species (ROS) production, complemented by cell-free DPPH radical scavenging assays. Fullerenol was taken up by neutrophils in a concentration- and time-dependent manner. No significant cytotoxicity was observed up to 100 μg/mL, while viability declined at 200 μg/mL. Phagocytosis of opsonized *E. coli* was preserved at lower concentrations, though a statistically significant negative correlation with fullerenol concentration was detected at higher doses. In cell-free assays, fullerenol scavenged DPPH radicals with an EC_50_ of 48.90 ± 10.02 μg/mL, exhibiting slower kinetics than Trolox or ascorbic acid. Critically, fullerenol suppressed intracellular ROS production by >33% at 50 μg/mL following PMA stimulation of neutrophils. These findings demonstrate that fullerenol C_60_(OH)_24_ combines potent intracellular antioxidant activity with a favorable neutrophil safety profile, supporting its potential application in oxidative stress-related conditions.

## 1. Introduction

In recent decades, the scientific community has directed a lot of attention toward finding ways of using nanomaterials in medicine. This interest stems primarily from the fact that such materials often have the potential to exhibit unique physicochemical properties, making them attractive candidates for biomedical applications. Major classes of nanoparticles considered in this context include quantum dots, dendrimers, polymers, carbon nanotubes, organic nanoparticles, metallic nanoparticles, liposomes, nanogels, and peptide-based nanoparticles [1]. As of 2025, about 500 nanoparticle-based drug formulations have reached clinical trials [2]. The recent successes of this approach include liposome-based COVID-19 vaccines and different types of polymeric nanoparticles used for drug delivery [3].

Carbon-based nanomaterials have been used in a very limited number of clinical trials. Despite this, they have been a subject of many scientific studies, possibly owing to their structural diversity, which in turn gives rise to a wide range of physical properties. This group of materials includes fullerenes, carbon nanotubes, graphene and nano-diamonds [4].

Fullerenes were discovered in 1985 by Kroto et al. [5]. A fullerene molecule is a closed cage made up entirely of carbon atoms [6] with a diameter of 0.71 nm [7]. Many applications of fullerene in engineering have been proposed [8,9]; however, due to their extremely low water solubility of 1.3 × 10^−11^ mg/mL [10] the use of this material in biological systems is restricted. Different fullerene derivatives have been synthesized to allow their use in aqueous media [11] with polyhydroxylated fullerene (also called fullerenol) being one of the most widely utilized.

Multiple uses of fullerenol nanoparticles in relation to biological systems have been proposed, including their application as antiviral [12,13], antitumor [14,15] and biovisualization agents [16,17]. At the same time, fullerenol is known to be a potent antioxidant [18], which allows to apply it for treatment of various pathological conditions related to oxidative stress. In that regard, fullerenol has been shown to mitigate doxorubicin-mediated heart damage in vivo [19] and prevent ethanol-induced liver damage [20]. Another intriguing application of fullerenol connected to its inherent antioxidant activity is the ability of these nanoparticles to mitigate damage induced by ionizing radiation as has been demonstrated in a radiation-induced dermatitis mouse model [21] and various other experiments [22], some of which utilized fullerenol derivatives such as metallofullerenols [23].

Fullerenol therefore possesses several properties that collectively make it an attractive candidate for biomedical development. Its potent free-radical scavenging capacity [24] combined with its high water solubility compared to unmodified fullerene [25], and its demonstrated efficacy in multiple animal models [26], provide a compelling basis for further investigation. At the same time, a number of challenges complicate translation of these findings. The physicochemical properties of fullerenol, including its degree of hydroxylation, aggregation state, surface charge, and the presence of synthesis-related impurities, may vary across preparations and influence biological activity [27], making direct comparisons between studies difficult. The absence of standardized synthesis and characterization protocols therefore represents a significant obstacle to the consistent and reproducible evaluation of fullerenol’s biomedical potential.

A further consideration that has received limited attention is the interaction of fullerenol with the immune system. It is well established that nanomaterials can trigger immune responses upon entry into a biological system, and that the nature of these responses—whether stimulatory or suppressive—is highly dependent on material properties such as size, surface chemistry, and functionalization [28]. Several studies have reported immunomodulatory effects of fullerene derivatives, including modulation of cytokine production and effects on phagocytic cell populations [14], though findings have been inconsistent across experimental systems. Critically, a substantial part of available evidence derives from in vivo models or experiments employing mixed-cell populations, in which the contributions of individual cell types cannot be readily distinguished. A precise, population-specific characterization of fullerenol’s immunological effects is nonetheless essential for rigorous safety evaluation and for the meaningful interpretation of more complex in vivo findings.

Despite the number of suggested applications, data on the cytotoxicity of fullerenol remains limited. Many studies using a wide array of methods for the assessment of cell viability have concluded that these nanoparticles lack cytotoxicity [15,29,30,31]. However, certain reports show that they may exhibit toxicity to some cell types, such as human umbilical cord endothelial cells [32,33] or monocytes [34], although it is unclear whether the observed toxicity is dependent on the type of cell, method of fullerenol synthesis, presence of impurities or other factors. Moreover, the effect of fullerenol on the functional activity of different cell types is not well characterized.

Among the cellular components of the immune system, neutrophils are particularly relevant to the safety evaluation of candidate nanomaterials. They represent the most abundant leukocyte population in peripheral blood and serve as central effectors of innate immunity, providing the first line of cellular defense against pathogens through phagocytosis and the generation of reactive oxygen species (ROS) [35]. Upon encountering foreign particulate matter, neutrophils may become activated, potentially triggering an inflammatory cascade that could either be harnessed therapeutically or represent an unintended adverse effect. Despite this, fullerenol’s effects on neutrophil viability and functional activity have not been systematically characterized. Given that neutrophil dysfunction, whether in the form of impaired pathogen clearance or dysregulated ROS production, carries significant clinical consequences, this gap in knowledge represents a meaningful limitation in the current understanding of fullerenol’s biological safety profile. It is well known that the immune system plays a major role in our body’s response to nanomaterials with some materials able to stimulate or suppress immune reactions [36], which may lead to undesired consequences preventing the use of a given material. Hence, this article focuses on investigating the effect of fullerenol nanoparticles on human neutrophils, assessing both cell viability, their phagocytic activity and ROS generation.

## 2. Materials and Methods

### 2.1. Materials and Equipment

The flow cytometer CytoFLEX S (Beckman Coulter, Brea, CA, USA) was used for sample analysis using CytExpert 2.4 software. The Primo Star light microscope (Carl Zeiss, Oberkochen, Germany) and Neubauer improved bright-line chamber (BRAND GMBH + CO KG, Wertheim, Genramy) were used for cell counting. The following reagents were used: Zombie Aqua (BioLegend, San Diego, CA, USA), Annexin V FITC (BioLegend, USA), Annexin V Binding Buffer (BioLegend, USA), Ficoll (Dia-M, Moscow, Russia), Bovine Serum Albumin (Miltenyl Biotec, Bergisch Gladbach, Germany), Sodium Azide (Dia-M, Russia), Lithium Heparin Blood Collection Tubes (MiniMed, Moscow, Russia), Dulbecco’s Phosphate-Buffered Saline (Paneco, Moscow, Russia), RPMI1640 w/o phenol red (Gibco, USA), anti-CD66b-PE antibodies (R&D Systems, Minneapolis, MN, USA), IL-8 ELISA kit (product number A-8762, Vector-Best, Novosibirsk, Russia), TNF-alpha ELISA kit (product number A-8756, Vector-Best, Russia), DMSO (PanReac Applichem, Barcelona, Spain), human normal serum-derived immunoglobulin (Microgen, Moscow, Russia), dihydrorhodamine 123 (Sigma-Aldrich, St. Louis, MO, USA), fluorescein isothiocyanate isomer I (Sigma-Aldrich, USA), phorbol 12-myristate 13-acetate (Sigma-Aldrich, USA), ascorbic acid (Vekton, Saint Petersburg, Russia), Trolox (MedChemExpress LLC, Monmouth Junction, NJ, USA), 2,2-diphenyl-1-picrylhydrazyl (Sigma-Aldrich, USA).

The CCM (complete culture medium) used in this study for cell cultivation consisted of RPMI-1640 (Gibco, Waltham, MA, USA) supplemented with 2 mM L-glutamine (Capricorn, Ebsdorfergrund, Germany), 100 units/mL penicillin, 0.1 mg/mL streptomycin, 2.5 µg/mL amphotericin B (BI, Kibbutz Beit Haemek, Israel), and 10% heat-inactivated fetal bovine serum (Capricorn, Germany).

### 2.2. Fullerenol Nanoparticles

Fullerenol C_60_(OH)_24_ was obtained from “Modern Synthesis Technology”, Russia (product number MST-WS60-Bio). The nanoparticles used in this study have been characterized previously [37]. Nanoparticle clusters in water have a volume-based mean particle diameter of 186 nm according to DLS measurements. The presence of no contaminants was identified as a result of UV/Vis and IR spectrophotometric analysis and the endotoxin content was measured to be 1.2 × 10^−3^ EU/μg for dry fullerenol using an LAL test.

### 2.3. Neutrophil Isolation

The study was conducted in accordance with the World Medical Association Declaration of Helsinki (2000) and the Protocol of the Council of Europe Convention on Human Rights and Biomedicine (1999). Approval for the use of peripheral blood samples was obtained from the Ethics Committee of the Institute of Ecology and Genetics of Microorganisms, Ural Branch of the Russian Academy of Sciences (IRB00010009), on 22 May 2024. Informed consent was obtained from all participants.

The neutrophils used in this study were isolated from heparinized peripheral blood of healthy donors (18–25 years old females). The blood was collected into tubes containing lithium heparin and diluted two-fold with a 1:1 DPBS:RPMI1640 mixture. The cells were isolated by centrifugation at 400 g for 40 min (with acceleration and deceleration settings set to 0 to preserve the gradient structure) using a double-density Ficoll–Verografin gradient (1.077 g/cm^3^ and 1.119 g/cm^3^). Neutrophil rings were collected and the cells were washed twice with a 1:1 DPBS:RPMI1640 mixture using centrifugation (at 350 g and 250 g for 20 min). After isolation, neutrophils were resuspended in the CCM. An aliquot was taken and the neutrophil concentration was determined using a counting chamber after the sample was diluted 1:19 in 5% acetic acid to lyse residual red blood cells.

### 2.4. Cell Viability and Fullerenol Sorption Assay

Neutrophils were seeded into the wells of a 96-well cell culture plate at 200,000 cells/well. Fullerenol nanoparticles were added to final concentrations of 0.25, 0.5, 2.5, 5, 12.5, 25, 50, 100 and 200 µg/mL. Wells containing cells with no added nanoparticles were used as a negative control. The cells were incubated in the presence of fullerenol for 24, 48, and 72 h in a humidified CO_2_ incubator (5% CO_2_, 37 °C). CD66b is a surface cell marker which is constitutively expressed by neutrophils [38] and was used by us for gating during flow cytometry. Zombie aqua (ZA) is a fluorescent live-dead dye which is often used in flow cytometry for viability determination [39,40]. At the end of the incubation period, the cells were stained with Zombie Aqua (λ_ex_ = 405 nm, bandpass filter 525 ± 20 nm) in the dark for 30 min. After washing with 2 mL of staining buffer (350 g, 10 min), they were then stained with anti-CD66b-PE (λ_ex_ = 488 nm, bandpass filter 585 ± 21 nm) antibodies for 30 min in the dark. Following staining, the cells were washed once again and analyzed using flow cytometry.

**Figure 1:**
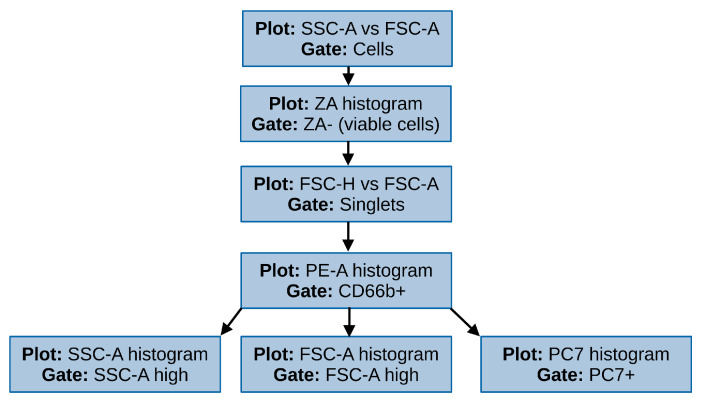
The gating strategy used for the assessment of fullerenol sorption by neutrophils.

The results of different studies highlight the ability of fullerenol to interact with cells in various ways, including both passive diffusion and active transport across the cell membrane as well as adhesion to its surface [41]. No conclusive experimental evidence has so far been presented as to which mode of interaction is the primary in terms of either the quantity of fullerenol engaged in an interaction nor in terms of its relevance for the observed biological effects of the nanoparticles. Since flow cytometry does not allow us to differentiate between internalization of nanoparticles and their adsorption on the cell surface, when describing the observed interaction, we will use the term “sorption” meaning either internalization or adherence. Fullerenol sorption was evaluated based on fluorescence of the nanoparticles, which was measured in the phycoerythrin-cyanine 7 (PE-Cy7, PC7) channel (λ_ex_ = 488 nm; bandpass filter: 780 ± 30 nm). Additionally, cell granularity and size were used for the evaluation of fullerenol sorption (Figure 1, Figure S1).

### 2.5. Apoptosis Assay

Neutrophils were seeded into the wells of a 96-well cell culture plate at 200,000 cells/well. Fullerenol nanoparticles were added to final concentrations of 0.25, 0.5, 2.5, 5, 12.5, 25, 50, 100 and 200 µg/mL. Wells containing cells with no added nanoparticles were used as a negative control. The cells were incubated in the presence of fullerenol for 24, 48, and 72 h in a humidified CO_2_ incubator (5% CO_2_, 37 °C). Following incubation, the cells were harvested into polypropylene tubes and washed with protein-free DPBS (350× *g*, 10 min; acceleration and brake set to 7). The cells were then stained with Zombie Aqua for 30 min in the dark at room temperature. After washing with DPBS supplemented with 10% BSA and 0.1% sodium azide (350× *g*, 10 min at 4 °C; acceleration and brake set to 7), the cells were stained with Annexin V-FITC in Annexin V binding buffer and incubated for 15 min at room temperature. Finally, Annexin V binding buffer was added, and the samples were placed on ice for flow cytometry analysis. The percentages of ZA^−^AnnV^+^ (early apoptotic) and ZA^+^AnnV^+^ (late apoptotic/necrotic) cells were determined. The neutrophil population was gated based on light-scatter characteristics. To determine the boundary of the negative population, unstained samples and FMO controls were used.

### 2.6. Preparation of FITC-Labeled Bacteria

The *Escherichia coli* strain K-12 at a concentration of 1 × 10^8^ cells/mL was opsonized for 1 h at 37 °C in the presence of polyclonal serum-derived human IgG (5 mg/mL). The bacteria were then washed twice to remove unbound immunoglobulins by centrifugation at 4500× *g* for 10 min in DPBS. Subsequently, the opsonized bacteria were labeled by adding a freshly prepared FITC solution in DMSO (1 mg/mL) to a final FITC concentration of 100 µg/mL. Staining was carried out for 1 h at 37 °C. Upon completion of staining, the cells were washed twice with DPBS to remove excess dye (4500× *g*, 10 min).

### 2.7. Phagocytosis Assay

Fullerenol nanoparticles were suspended in phenol-red free RPMI1640 CCM. Neutrophils were seeded into plastic test tubes at a concentration of 4 × 10^6^ cells/mL (50 μL per tube). Subsequently, fullerenol nanoparticles were added to final concentrations of 0.25, 2.5, 5, 12.5, 50, 100 and 200 µg/mL, the tubes were sealed with a gas permeable membrane and the cells were incubated for 24 h in a CO_2_ incubator (5% CO_2_; 37 °C) in the phenol-red free RPMI1640 CCM. Then, an equal volume (100 µL) of FITC-labeled opsonized *E. coli* K12 was added to the tubes, and the mixture was incubated for an additional 30 min at 37 °C. At the end of the incubation period, all samples were placed on ice for 10 min, washed once with 2 mL of cold DPBS with 0.5% BSA and 0.1% sodium azide (350 g, 10 min), placed on ice again, and analyzed using a flow cytometer. To evaluate the uptake/adhesion of bacteria by the cells, the percentage of FITC-positive cells within the neutrophil gate was determined for each sample. A similar phagocytosis assay procedure has been used in other studies [42,43,44,45].

The correlation between the concentration of fullerenol and the fraction of *E. coli*-positive cells was assessed using Spearman rank correlation analysis, performed with the SciPy version 1.17.0 Python package. A one-sided test was performed to evaluate the hypothesis of a negative monotonic association between the two parameters. The Spearman correlation coefficient (ρ) and the corresponding *p*-value were calculated.

### 2.8. DPPH Assay of Antioxidant Activity

The antioxidant activity of fullerenol was determined using the DPPH (2,2-diphenyl-1-picrylhydrazyl) method. DPPH• is a stable radical that can be reduced by antioxidants to form the DPPH molecule. Upon reduction, the radical changes color, allowing the reaction progress to be monitored with spectrophotometry. The antioxidant activity of fullerenol was compared with two standards: Trolox and ascorbic acid. These compounds are widely used in antioxidant activity assays and serve as references for comparing the activities of different substances.

The analysis was performed in a 96-well flat-bottom plate. Each well was loaded with 100 µL of sample solution (fullerenol, Trolox, or ascorbic acid) at various concentrations, followed by the addition of 100 µL of a 150 mM DPPH• solution in ethanol. The plate was incubated in the dark and sealed with an airtight film to prevent ethanol evaporation. The optical density was measured every 30 min at a wavelength of 520 nm.

Since fullerenol absorbs light at 520 nm, the absorbance of a fullerenol solution of the same concentration without DPPH• was subtracted from the measured values during data processing. For Trolox and ascorbic acid, the optical density was corrected for the absorbance of water. The final values were expressed as a percentage of the optical density of the control DPPH• solution, to which no antioxidants were added.

The activities of fullerenol, ascorbic acid, and Trolox were compared using the EC_50_ parameter, defined as the concentration at which the optical density decreases by 50% relative to the absorbance of the reference DPPH solution. The EC_50_ values were calculated by fitting the measured absorbance values on the antioxidant concentration using the least squares method. It was observed that, in contrast to Trolox and ascorbic acid, fullerenol exhibited slower reaction kinetics compared to the reference substances. Therefore, when processing the results for fullerenol, the absorbance values obtained from the final two measurements (i.e., over the last 60 min) were used for the EC_50_ calculation.

### 2.9. ROS Production Assay

Peripheral blood neutrophils were isolated from peripheral blood using the method described previously. The cells were washed, counted, and resuspended in phenol red-free RPMI 1640 medium to a final concentration of 2 × 10^6^ cells/mL. For intracellular detection of reactive oxygen species (ROS) by flow cytometry, the cells were pretreated with dihydrorhodamine 123 (DHR123), which passively diffuses across cell membranes and is oxidized to fluorescent rhodamine 123. Neutrophils were incubated with DHR123 at a concentration of 2.5 µM for 10 min at 37 °C. The cells were then washed to remove excess DHR123 by centrifugation in DPBS at 250× *g* for 10 min.

DHR123-treated neutrophils were transferred into sterile 5 mL polypropylene tubes in culture medium to a final concentration of 1 × 10^6^ cells/mL. Fullerenol was then added to achieve final concentrations of 10 and 50 µg/mL. The cells were incubated for 90 min at 37 °C in a humidified CO_2_ incubator with 5% CO_2_. Cell cultures without fullerenol served as controls. To stimulate ROS production, phorbol 12-myristate 13-acetate (PMA) was added to each tube at a final concentration of 20 nM. After 90 min, the samples were centrifuged to remove the supernatant and perform fixation by adding 350 μL of a 1% paraformaldehyde solution in DPBS. The samples were then analyzed using the CytoFLEX S flow cytometer.

Rhodamine 123-positive events were detected in the FITC fluorescence channel of the flow cytometer (488 nm excitation laser, 525 ± 20 nm bandpass filter). Dead and apoptotic cells were excluded by gating on FSC/SSC dot plots, and doublets were excluded using FSC-A/FSC-H plots. For each sample, the mean fluorescence intensity (MFI) was determined as the median fluorescence intensity in the rhodamine 123 channel.

### 2.10. Assessment of Cytokine Production

To assess the effect of fullerenol nanoparticles on peripheral blood neutrophils, cytokine levels—tumor necrosis factor alpha (TNF-alpha) and interleukin-8 (IL-8)—were measured in culture supernatants after treatment with fullerenol for 24 or 72 h. To collect the supernatants for analysis, the cell culture plates were centrifuged at 350 g for 10 min. Cytokine concentrations were determined by enzyme-linked immunosorbent assay (ELISA) using commercial IL-8 and TNF-alpha kits, in accordance with the manufacturer’s instructions.

### 2.11. Statistical Analysis

Pandas [46], SciPy [47] and Vega-Altair [48] were used for data analysis and visualization. Statistical comparisons were performed with Dunnett’s test. Differences between control and experimental groups with the *p*-value of less than 0.05 were considered significant.

## 3. Results

### 3.1. Sorption of Nanoparticles

Previous studies involving other types of materials, such as gold [49] or TiO_2_ [50], have demonstrated that the uptake of nanoparticles by mammalian cells can be observed by changes in the forward and side scattering of light in flow cytometry assays. Based on those findings, we decided to evaluate whether this strategy can be applied in regard to fullerenol.

Assessment of neutrophil granularity and cell size revealed no statistically significant differences (Figure S2) between cultures treated with different concentrations of fullerenol and untreated controls. Therefore, we conclude that these parameters cannot be used to detect fullerenol sorption by human neutrophils and a different approach is required. In this study, we used fluorescence in the PC7 channel to detect fullerenol sorption as these nanoparticles exhibit autofluorescence at λ_ex_ = 430 nm and an emission maximum at around 560 nm as we have demonstrated in a previous study [37].

**Figure 2:**
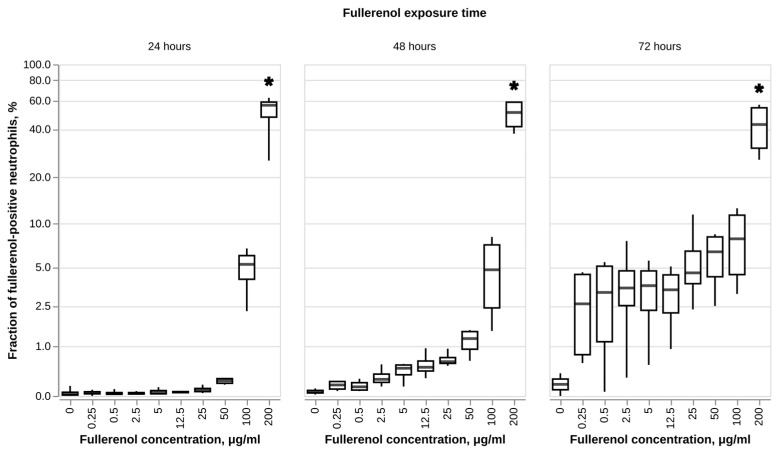
Sorption of fullerenol C_60_(OH)_24_ by human neutrophils based on autofluorescence in the PC7 channel. N = 3. Median and IQR values are shown. Statistically significant differences (Dunnett’s test) between each test group and negative control at every time point are marked with * (*p*-value < 0.05).

Evaluation of fullerenol sorption based on cell autofluorescence in the PC7 channel indicates that the extent of nanoparticle sorption is both concentration- and time-dependent (Figure 2). These results are in good agreement with various prior publications, which show that fullerenol nanoparticles can be internalized by mammalian cells [51,52,53]. More specifically, regarding the immune cells, fullerenol internalization was demonstrated by Lichota et al. for human peripheral blood mononuclear cells [54]. Previously, using more sensitive methods which allow us to determine the locations of nanoparticles in relation to cells (such as confocal and transmission electron microscopy), other authors have observed that fullerenol nanoparticles are able to enter the cytoplasm and nucleus of mammalian cells [51,54]. However, some pieces of evidence also point to the ability of fullerenol to adhere to cellular membranes [55].

The concentration-dependent pattern of fullerenol sorption observed in our study aligns with prior experimental data showing a direct correlation between the amount of fullerenol bound to the cell membrane and its concentration in the surrounding medium [55]. This relationship is also consistent with in silico data suggesting that fullerenol molecules with a sufficiently high number of hydroxyl groups tend to adhere to the membrane surface rather than penetrate the lipid bilayer [56], implying that sorption at the membrane is a likely component of the signal detected in our assay.

The time-dependent increase in fluorescence signal is consistent with findings by Lichota et al., who demonstrated that internalization of fullerenol by peripheral blood mononuclear cells was directly proportional to both the particle concentration and the incubation time [54]. Those authors also observed that with longer incubation times, accumulation of fullerenol inside the cells becomes predominant over its adsorption on the membrane surface. Endocytosis appears to be the most likely mechanism of fullerenol uptake into cells, as suggested by studies showing rapid accumulation of radiolabeled fullerenol by CHO cells and its localization within endosomal compartments in TEM sections [51,57].

It should be noted that the relative contributions of surface adsorption and active internalization to the total PC7 fluorescence signal cannot be fully distinguished by flow cytometry alone, since this method integrates fluorescence from nanoparticles both at the membrane surface and within the cell interior. Methods that allow direct localization of nanoparticles in relation to cells, such as confocal and transmission electron microscopy, would be required to more precisely characterize the spatial distribution of fullerenol sorption in human neutrophils.

### 3.2. Effect of Fullerenol on Neutrophil Viability

**Figure 3:**
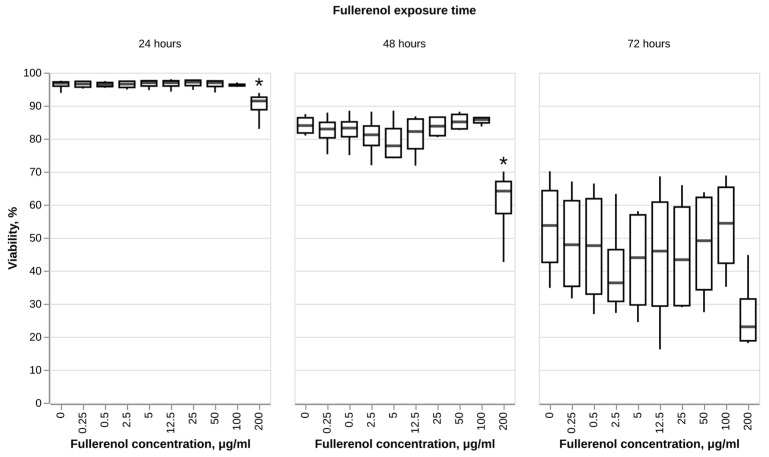
Effect of fullerenol C_60_(OH)_24_ on viability of human neutrophils. N = 3. Median and IQR values are shown. Statistically significant differences (Dunnett’s test) between each test group and negative control at every time point are marked with * (*p*-value < 0.05).

In this study, fullerenol did not show any cytotoxic effect toward human neutrophils when used at concentrations of up to 200 μg/mL (Figure 3). At the concentration of 200 μg/mL a statistically significant decrease in cell viability was observed when cells were incubated in the presence of the nanoparticles for 24 and 48 h. In the group exposed to the nanoparticles for 72 h, no significant viability decrease was registered. However, the viability of all groups regardless of fullerenol concentration decreased after 72 h of exposure, which can be attributed to the short life span of neutrophils—generally considered to be less than 5 days in vivo [58].

**Figure 4:**
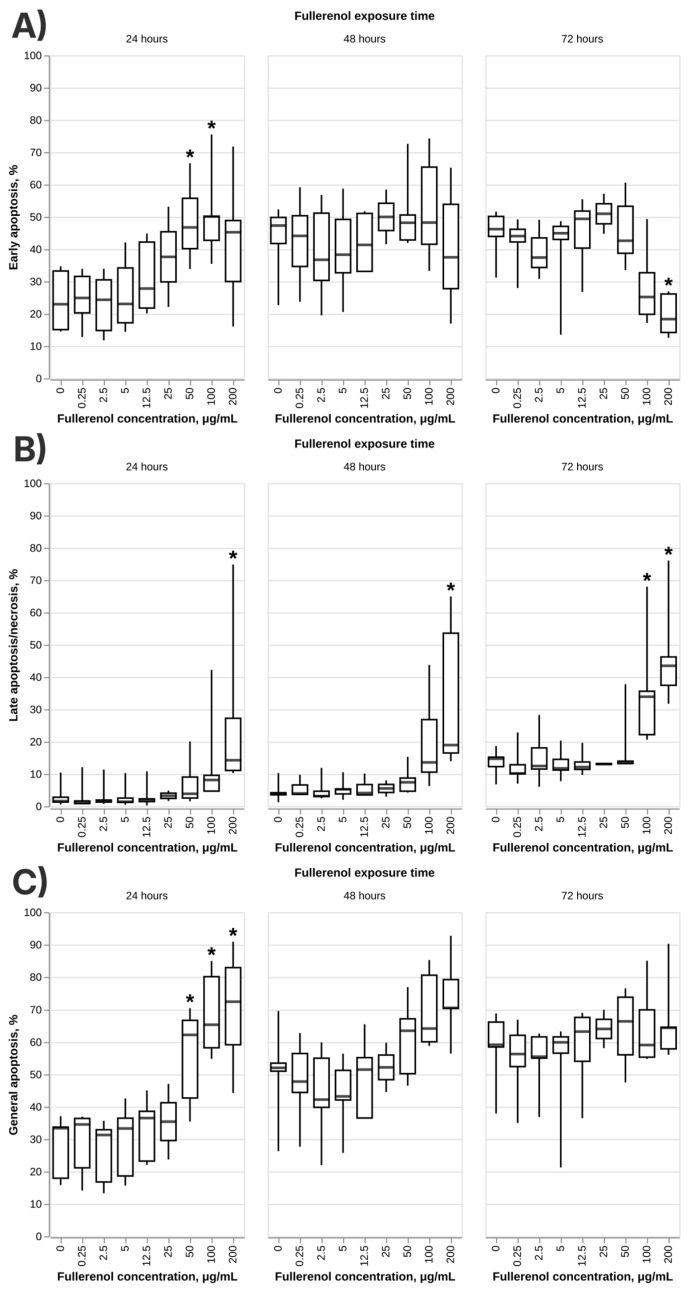
Effect of fullerene C_60_(OH)_24_ on early (**A**), late (**B**) and general (**C**) apoptosis of normal human neutrophils after 24–72 h incubation with the nanoparticles. N = 5. Median and IQR values are shown. Statistically significant differences (Dunnett’s test) between each test group and negative control at every time point are marked with * (*p*-value < 0.05).

An increase in the percentage of apoptotic cells also supports the findings of the negative effect of fullerenols on the viability of neutrophils at higher nanoparticle concentrations (Figure 4).

Previous studies demonstrate that fullerenol generally does not exert cytotoxic effects on mammalian cells in the range of concentrations used in this study [31,59,60]. However, a few experiments have also shown that fullerenol might be cytotoxic [57,61] at concentrations of 100–200 μg/mL. It is not known at this point which factors determine the cytotoxicity profile of these nanoparticles, but it is reasonable to assume that specific traits of certain cell lines might be an important factor. For example, two independent studies demonstrated the toxicity of fullerenol to human umbilical cord endothelial cells [32,33]. In light of the theory of the cell line-specific toxicity of fullerenol, this article for the first time demonstrates the toxic effect of these nanoparticles to human neutrophils.

The mechanisms underlying the cytotoxic effects observed at 200 μg/mL in our study are potentially multifactorial. Several potential mechanisms of fullerenol-induced cytotoxicity have been described in the literature. Fullerenol has been shown to bind to actin in the cellular cytoskeleton, impairing its function, with incubation of animal cell lines with fullerenol leading to a decrease in cell membrane stiffness [62]. Mitochondrial dysfunction represents another potential mechanism as fullerenol C_60_ has been reported to alter the ultrastructure of isolated mitochondria, increasing the permeability of the inner membrane to K^+^ and H^+^ ions and disrupting the transmembrane potential [63]. Additionally, the role of nanoparticle synthesis-related impurities as a potential source of cytotoxicity should be considered, as toxic impurities introduced during synthesis have been proposed as a contributing factor in other fullerenol studies [64]. It is worth noting that fullerenol C_60_(OH)_24_ is capable of binding to DNA in aqueous solution at physiological pH, specifically to its phosphate backbone [65], though studies on CHO-K1 cells demonstrated no genotoxicity across a broad concentration range [66], and a similar absence of genotoxic effects on peripheral blood mononuclear cells has been reported [67].

Multiple factors are likely contributing jointly to determining the cytotoxicity profile of fullerenol, including the physicochemical properties of the nanoparticles (size, surface charge, and presence of synthetic impurities), characteristics of the exposure conditions (medium composition, protein content, and nanoparticle concentration), and cell-specific properties such as metabolic and phagocytic activity. The cytotoxic effect in our study appears to be confined to the highest tested concentration (200 μg/mL) and was not observed at 100 μg/mL or below, which is consistent with the broader literature demonstrating the good biocompatibility of fullerenol across a wide concentration range.

### 3.3. Effect of Fullerenol on Phagocytosis

**Figure 5:**
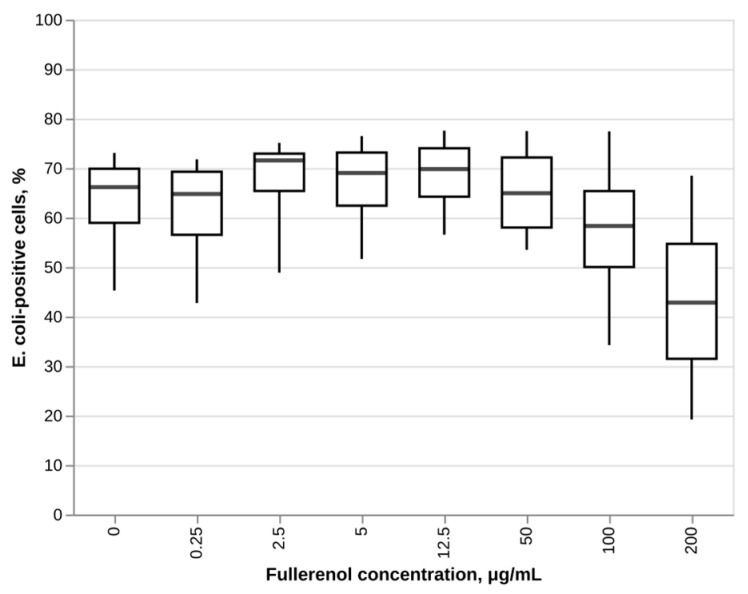
Effect of fullerenol C_60_(OH)_24_ on phagocytosis of *E. coli* by human neutrophils after a 24 h treatment with fullerenol and a 30 min incubation with FITC-labeled opsonized *E. coli*. N = 4. Median and IQR values are shown. No statistically significant differences (Dunnett’s test) between test groups and negative control were found (*p*-value < 0.05).

Fullerenol C_60_(OH)_24_ did not have a statistically significant effect on the phagocytosis of *E. coli* by human neutrophils (Figure 5). The obtained results are contrary to our initial hypothesis that treatment with fullerenol would impair neutrophils’ ability to engulf bacteria as a result of the ability of these nanoparticles to bind to the actin cytoskeleton which was demonstrated previously [68,69].

At the same time, a negative correlation between the fullerenol concentration and the fraction of *E. coli* positive neutrophil cells can be observed at high fullerenol concentrations (12.5–200 μg/mL). A Spearman rank correlation analysis revealed a statistically significant negative monotonic association between these parameters (rho-value = −0.546, *p* = 0.014). These findings indicate that the observed lack of statistically significant differences in pairwise comparisons may be due to the high level of variance within the measurements. Therefore, it is plausible that given a larger sample size, a statistically significant effect of fullerenol on phagocytosis of human neutrophils could be demonstrated.

### 3.4. Evaluation of the Antioxidant Activity of Fullerenol in a Cell-Free System

**Figure 6:**
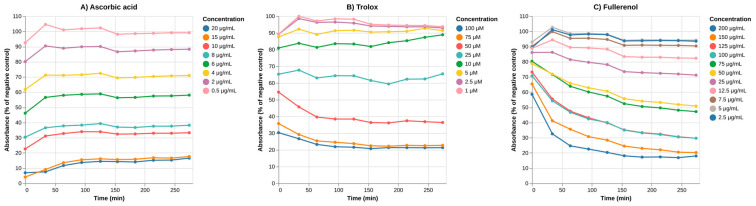
Change in absorbance during a DPPH-based assay of fullerenol antioxidant activity in comparison with Trolox and ascorbic acid (kinetic measurement). N = 3. Mean values are shown.

The obtained data demonstrate pronounced antioxidant activity of fullerenol C_60_(OH)_24_ (Figure 6). The inclusion of standard antioxidants in the study allows us to conduct a comparison between the activities of fullerenol and reference compounds (Trolox and ascorbic acid). Specifically, to perform a quantitative comparison we calculated the EC_50_ values for each substance (Table 1) based on the relationship between the concentration of the antioxidant and its ability to facilitate the reduction of the DPPH radical measured as the reduction in the optical absorbance, to which DPPH• contributes (Figure S3).

It is worth noting that, unlike Trolox and ascorbic acid, fullerenol exhibited slower reaction kinetics with DPPH (Figure 6). This characteristic may be advantageous for its medical application as an antioxidant, as the slower reaction kinetics may provide prolonged protection against reactive oxygen species generated by the administration of chemotherapeutic agents or radiation exposure.

The antioxidant activity of fullerenol, expressed as Trolox equivalents, was found to be close to 1, whereas its molecular weight is more than four times that of Trolox (Table 1). This indicates that a higher mass of fullerenol is required to achieve an equivalent antioxidant effect. Given that fullerenol consists of nanoparticles prone to aggregation, this could be interpreted as a limitation; however, its significance depends primarily on the toxicity profile of fullerenol.

### 3.5. Effect of Fullerenol on ROS Production in Vitro

**Figure 7:**
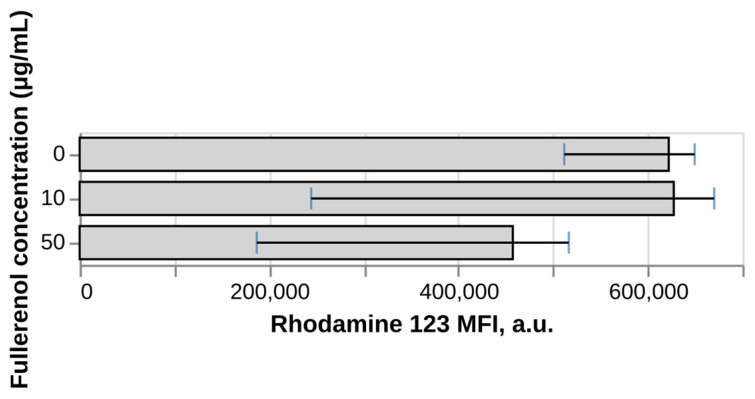
Effect of fullerenol on intracellular ROS levels in neutrophils. N = 4. Median and IQR are shown. No statistically significant differences (Dunnett’s test) between test groups and negative control were found (*p*-value < 0.05).

As can be seen in Figure 7, fullerenol C_60_(OH)_24_ suppresses intracellular production of ROS at a concentration of 50 μg/mL by more than 33%; however, due to the high level of variance, no statistically significant differences were found between the experimental groups and the control. While a trend toward attenuation of oxidative stress is observed, the absence of statistical significance does not allow us to make a definitive conclusion regarding the scavenging capacity of the nanomaterial at this dosage. The observed tendency for decreased ROS levels may suggest that fullerenol has the potential to intercept free radicals—such as superoxide anions (O2•–) and hydroxyl radicals (•OH)—though further investigation with a larger sample size would be required to confirm this effect.

Previously, the ability of fullerenol to exhibit antioxidant activity has been demonstrated in cell-free systems using DPPH• [70] and the oxidation of methyl linoleate [71] as well as using various cell lines. For example, Xu et al. have shown the cytoprotective effect of micelles containing fullerenol nanoparticles when cells are exposed to doxorubicin [72]. However, existing studies put little emphasis on the distinction between the ability of fullerenol to exert its protective effect within or outside cells. Importantly, since DHR123 is localized within cells, our study demonstrates that fullerenol has shown a promising potential to exert its antioxidant activity within live cells, allowing it to provide protection against oxidative stress not just in the extracellular environment.

### 3.6. Effect of Fullerenol on Cytokine Production

**Figure 8:**
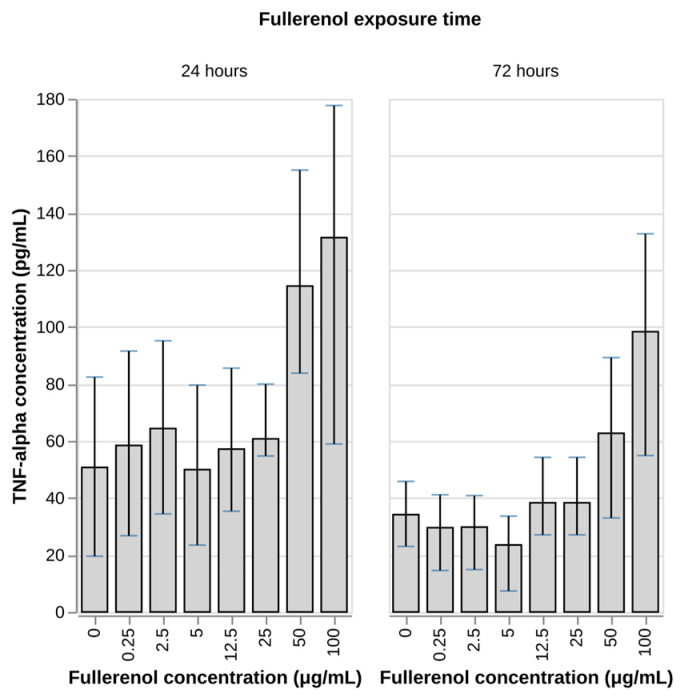
Concentration of TNF-alpha after 24 h and 72 h incubation of human peripheral blood neutrophils with fullerenol nanoparticles. N = 4 for 24 h; N = 3 for 72 h. Mean values and IQR are shown. No statistically significant differences (Dunnett’s test) between test groups and negative control were found (*p*-value < 0.05).

**Figure 9:**
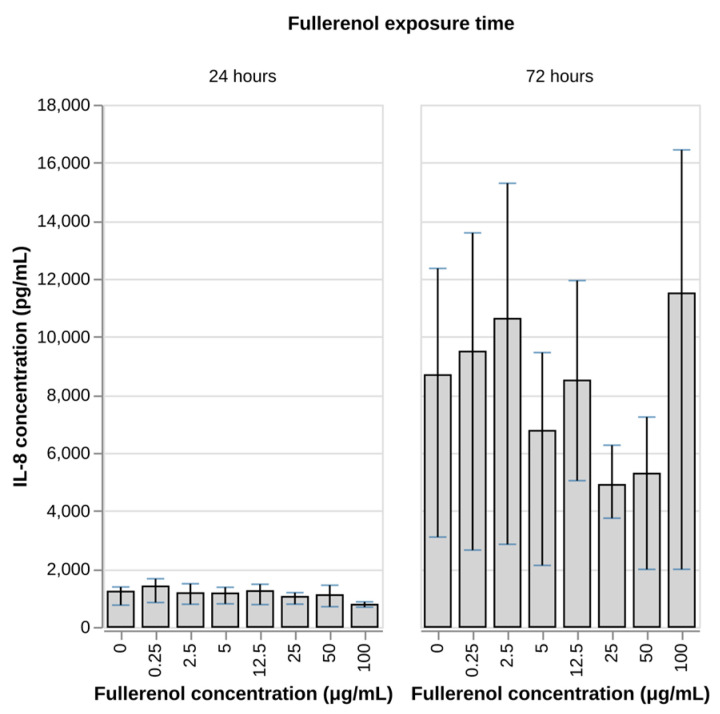
Concentration of IL-8 after a 24 h incubation of human peripheral blood neutrophils with fullerenol nanoparticles. N = 4 for 24 h; N = 3 for 72 h. Mean values and IQR are shown. No statistically significant differences (Dunnett’s test) between test groups and negative control were found (*p*-value < 0.05).

In this study, we evaluated the effect of fullerenol on the production of IL-8 and TNF-alpha by the neutrophil cells. IL-8 is a key mediator of inflammation whose secretion is enhanced under conditions of oxidative stress while TNF-alpha is a proinflammatory cytokine that plays an active role in immune and inflammatory responses. During exposure of neutrophils to fullerenol at concentrations of 50 and 100 µg/mL for 24 and 72 h, a tendency toward increased TNF-alpha levels in culture supernatants was observed, whereas IL-8 concentrations remained unchanged (Figure 8 and Figure 9).

Thus, the obtained results indicate a proinflammatory effect of fullerenol on neutrophils, manifested by selective stimulation of TNF-alpha secretion without any effect on IL-8 production. The tendency toward increased TNF-alpha levels observed after 24 and 72 h of incubation with fullerenol (50–100 µg/mL) suggests a dose- and time-dependent activation of proinflammatory pathways in neutrophils.

The observed apparent increase in the concentration of TNF-alpha is consistent with our data on the frequency of apoptosis of neutrophils after fullerenol exposure, as the concentration-dependent correlation between increased TNF-alpha secretion (50–100 µg/mL) and elevated apoptosis rates at the highest concentrations (200 µg/mL) suggests that TNF-alpha may be a mechanistic driver of the apoptotic effects observed. Given that TNF-alpha is a well-established inducer of the extrinsic apoptotic pathway [73,74] via caspase-8 activation, the cytokine elevation triggered by fullerenol exposure could directly contribute to the reduced cell viability and increased apoptosis detected at higher nanoparticle concentrations. This relationship underscores the complex immunomodulatory profile of fullerenol, wherein the same nanoparticle-induced inflammatory response may simultaneously mediate both immunostimulatory (TNF-alpha secretion) and cytotoxic (apoptosis) outcomes.

These results are consistent with other studies indicating that fullerenol exposure induces the secretion of proinflammatory cytokines in various cell types. For example, Tang et al. demonstrated stimulation secretion of TNF-alpha, IL-1beta and IL-6 by macrophages [75]. Stimulation of proinflammatory cytokine production has also been demonstrated during experiments involving in vivo mice [76] and rat [77] models. Overall, the presently available evidence strongly points toward the ability of fullerenol nanoparticles to induce proinflammatory response in immune cells, but the mechanisms of such activity are not well understood. Tang et al. demonstrated that this activity is dependent on the NFkB activation pathway [75]. Given the low endotoxin content of fullerenol nanoparticles used in this study as established with the LAL test [37], it is plausible to assume that the proinflammatory activity of fullerenol may be an intrinsic property of the nanoparticles and not a result of contamination with lipopolysaccharide.

## 4. Discussion

This study provides the first comprehensive characterization of the interaction between fullerenol C_60_(OH)_24_ nanoparticles and human neutrophils, addressing an important gap in the existing literature regarding the immunomodulatory effects of water-soluble fullerene derivatives toward specific immune cell lines in vitro. Our findings reveal a complex relationship that must be carefully considered when evaluating the potential biomedical applications of these nanoparticles.

The demonstrated concentration- and time-dependent sorption of fullerenol by neutrophils establishes that these nanoparticles can effectively interact with human immune cells. This was confirmed through fluorescence detection in the PC7 channel. The absence of significant cytotoxicity at concentrations up to 200 μg/mL, with only minor decreases in viability at the highest concentration after 24–48 h of exposure, aligns with previous reports of the general biocompatibility of fullerenol across various cell types. However, the observation of transient toxicity at 200 μg/mL warrants further investigation to determine whether this represents a genuine concentration-dependent effect or an artifact related to nanoparticle aggregation or cell-specific sensitivity.

The biocompatibility of fullerenol observed in the present study is consistent with findings reported for other immune cell types. For example, fullerenol C_60_(OH)_36_ did not significantly affect viability of PBMCs at concentrations of 50–150 mg/L over 24 h [54], and no reduction in the viability of primary macrophages, B cells or T cells was observed at concentrations up to 100 µM over 72 h [14]. For the same hydroxylation degree as the nanoparticles used herein, no cytotoxicity was observed in mouse peritoneal macrophages at concentrations up to 0.12 mg/mL, with a marked viability decrease only above this threshold [76]—a pattern broadly comparable to our observation of declining viability at 200 µg/mL. Notably, the absence of cytotoxicity does not preclude functional effects: elevated TNF-alpha was reported in macrophage and T-cell supernatants [14], and enhanced phagocytosis and production of IL-1beta, IL-6, and TNF-alpha in peritoneal macrophages was documented following fullerenol treatment without cytotoxicity [75]. Our finding of increased TNF-alpha secretion by neutrophils in the absence of significant cell death is therefore in line with this pattern. However, direct comparison across these studies is complicated by differences in hydroxylation degree, cell type, and species origin, and critically, several studies did not report endotoxin testing of their nanoparticle preparations, leaving open the possibility that some reported proinflammatory effects reflect contamination rather than intrinsic nanoparticle activity. In the present study, the proinflammatory effect was confirmed under endotoxin-controlled conditions, strengthening the conclusion that TNF-alpha stimulation is an intrinsic property of fullerenol C_60_(OH)_24_.

Notably, fullerenol treatment did not impair neutrophil phagocytosis of *E. coli*, contrary to our initial hypothesis based on previous reports of fullerene interaction with the actin cytoskeleton. This preservation of a critical neutrophil function suggests that fullerenol may be less disruptive to cytoskeletal dynamics than anticipated. However, the observed trend for the decrease in phagocytic activity at higher fullerenol concentrations suggests that may still be detected given a larger sample size.

Perhaps the most clinically significant finding is the dose- and time-dependent suppression of intracellular reactive oxygen species (ROS) production at 50 μg/mL. This antioxidant activity, demonstrated through DHR123 oxidation assays, suggests a potential free radical scavenging capacity of fullerenol within living neutrophils, though the absence of statistical significance due to high inter-donor variance calls for cautious interpretation. Given the central role of oxidative stress in numerous pathological conditions, including inflammatory diseases, neurodegenerative disorders, and ischemia–reperfusion injury, this intracellular antioxidant effect represents a promising therapeutic property that could be harnessed for the treatment of conditions characterized by excessive ROS production.

Simultaneously, our observation of increased TNF-alpha secretion without corresponding changes in IL-8 production reveals a complex immunomodulatory profile. The selective stimulation of proinflammatory TNF-alpha suggests that fullerenol can activate specific inflammatory pathways in neutrophils, possibly through NF-κB-dependent mechanisms as proposed by other researchers. This proinflammatory activity, which appears to be an intrinsic property of fullerenol rather than an artifact of endotoxin contamination, creates an intriguing paradox: the same nanoparticle that suppresses ROS may also stimulate certain aspects of the inflammatory response. This duality may have important implications for the use of fullerenol as an immunomodulatory agent, as the balance between antioxidant and proinflammatory effects could determine therapeutic outcomes in different clinical contexts.

Several methodological limitations should be considered when interpreting the results of this study. First of all, flow cytometry cannot distinguish between nanoparticle internalization and surface adsorption, necessitating use of the term “sorption” to encompass both. Small sample sizes (N = 3–5 donors) may have limited statistical power, particularly for detecting subtle effects. Additionally, fullerenol aggregation states were not systematically characterized, and the DPPH antioxidant assay may not fully represent complex antioxidant mechanisms in biological systems. The in vitro design using neutrophils from healthy donors limits generalizability to other populations or disease states. Furthermore, focusing on a single immune cell type provides a limited perspective on overall immunomodulatory effects, and the mechanistic pathways underlying the observed effects remain unelucidated.

In summary, this study establishes that fullerenol C_60_(OH)_24_ nanoparticles interact with human neutrophils in a concentration- and time-dependent manner, with a generally favorable cytotoxicity profile and a limited effect on phagocytic function but complex immunomodulatory properties that combine potent intracellular antioxidant activity with proinflammatory stimulation. The preservation of neutrophil function, coupled with a notable trend toward ROS scavenging capacity, supports the potential therapeutic utility of fullerenol in conditions characterized by oxidative stress. However, the proinflammatory effects underscore the need for careful risk–benefit assessment and dose optimization in clinical applications. A particular benefit of this work lies in its controlled in vitro design, which allowed us to isolate and characterize the direct effects of fullerenol on a single, well-defined immune cell population—human peripheral blood neutrophils—without the confounding influence of other cell types, systemic factors, or intercellular communication that would inevitably be present in vivo. Furthermore, the fullerenol preparation used in this study was thoroughly characterized with respect to both endotoxin content and heavy metal contamination, allowing us to attribute the observed proinflammatory effects—in particular the increase in TNF-alpha secretion—to the nanoparticle itself rather than to contaminants. This is a notable methodological distinction from several previously published studies on fullerenol and immune cells, in which endotoxin testing was not reported and the intrinsic nature of observed proinflammatory responses therefore remained uncertain. These findings provide a foundation for future investigations aimed at harnessing the beneficial properties of fullerenol while mitigating potential risks, ultimately advancing the development of safe and effective fullerenol-based nanomedicines.

## Figures and Tables

**Table 1:** EC_50_ values for ascorbic acid, Trolox and fullerenol C_60_(OH)_24_. Values are presented as mean ± SD. N = 3.

Substance	EC_50_	Antioxidant Activity (μM Trolox/μM Substance)
Ascorbic acid	6.54 ± 0.49 μg/mL	0.98
Trolox	37.89 ± 6.15 μM	1
Fullerenol C_60_(OH)_24_	48.90 ± 10.02 μg/mL	1.16

## Data Availability

The original contributions presented in this study are included in the article/Supplementary Materials. Further inquiries can be directed to the corresponding author.

## Supplementary Materials

The following supporting information can be downloaded at: https://www.mdpi.com/article/10.3390/nano16070405/s1, Figure S1: The gating strategy for the determination of highly granular and large neutrophils; Figure S2: Percentage of highly granular and large neutrophils after fullerenol treatment; Figure S3: Change in absorbance during a DPPH-based assay of fullerenol antioxidant activity in comparison with Trolox and ascorbic acid (end-point measurement).

## Abbreviations

The following abbreviations are used in this manuscript:

AnnV	Annexin V
BSA	Bovine serum albumin
CCM	Complete culture medium
DHR123	Dihydrorhodamine 123
DLS	Dynamic light scattering
DMSO	Dimethyl sulfoxide
DPBS	Dulbecco’s phosphate buffered saline
DPPH	2,2-diphenyl-1-picrylhydrazyl
ELISA	Enzyme-linked immunosorbent assay
FBS	Fetal bovine serum
FITC	Fluorescein isothiocyanate
FMO	Fluorescence minus one
FSC-A	Forward scatter-area
FSC-H	Forward scatter-height
IQR	Interquartile range
IR	Infrared
LAL	Limulus amebocyte lysate
MFI	Mean fluorescence intensity
PC7	Phycoerythrin-cyanine 7
PE	Phycoerythrin
PE-A	Phycoerythrin-area
PMA	Phorbol 12-myristate 13-acetate
ROS	Reactive oxygen species
SSC-A	Side scatter parameter-area
UV/Vis	Ultraviolet-to-visible
ZA	Zombie aqua
