# Supplementary material for: Effect of Fullerenol C60(OH)24 on Viability and Phagocytic Activity of Human Neutrophils
Source: Nanomaterials (Basel). 2026 Mar 27;16(7):405. doi: 10.3390/nano16070405 (PMC13075029; doi:10.3390/nano16070405)
Supplement: Supplementary file 1 [file nanomaterials-16-00405-s001.zip › Figure S2.pdf]

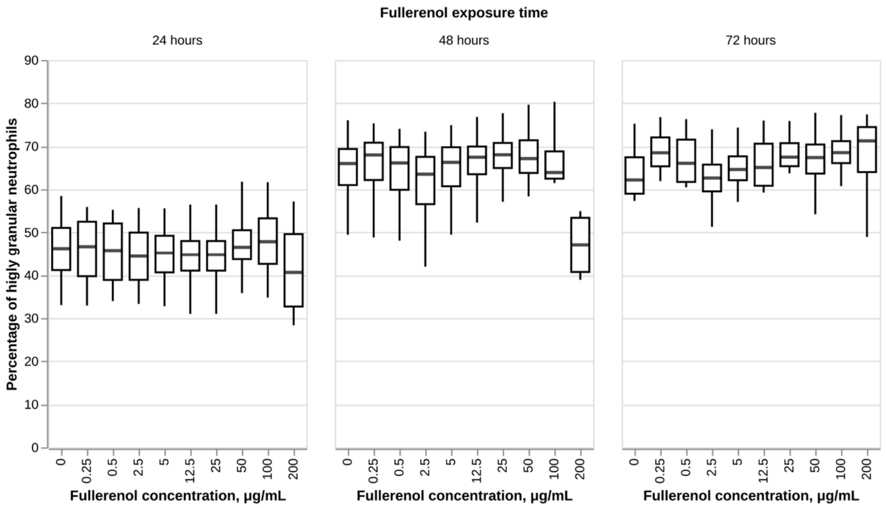

Figure S2.1. Percentage of highly granular cells (SSC high) in neutrophil cultures incubated for 24, 48, and 72 h with different concentrations of fullerenol $C_{60}(OH)_{24}$. N=4. Median and IQR are shown. Statistically significant differences (Dunnet's test) between each test group and negative control at every time point are marked with * (p-value < 0.05).

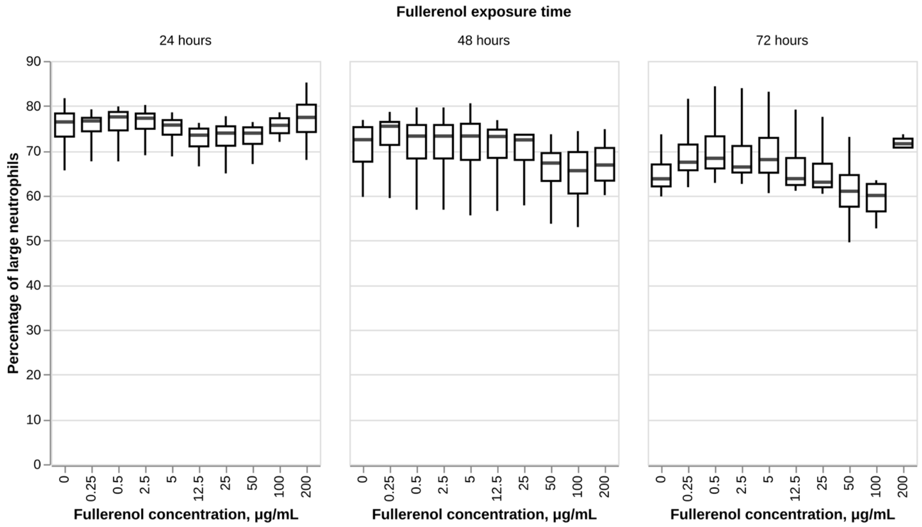

Figure S2.2. Percentage of large cells (FSC high) in neutrophil cultures incubated for 24, 48, and 72 h with different concentrations of fullerenol $C_{60}(OH)_{24}$. N = 4. Median and IQR are shown. Statistically significant differences (Dunnet's test) between each test group and negative control at every time point are marked with * (p-value < 0.05).
