# Supplementary material for: Effect of Fullerenol C60(OH)24 on Viability and Phagocytic Activity of Human Neutrophils
Source: Nanomaterials (Basel). 2026 Mar 27;16(7):405. doi: 10.3390/nano16070405 (PMC13075029; doi:10.3390/nano16070405)
Supplement: Supplementary file 1 [file nanomaterials-16-00405-s001.zip › Figure S3.pdf]

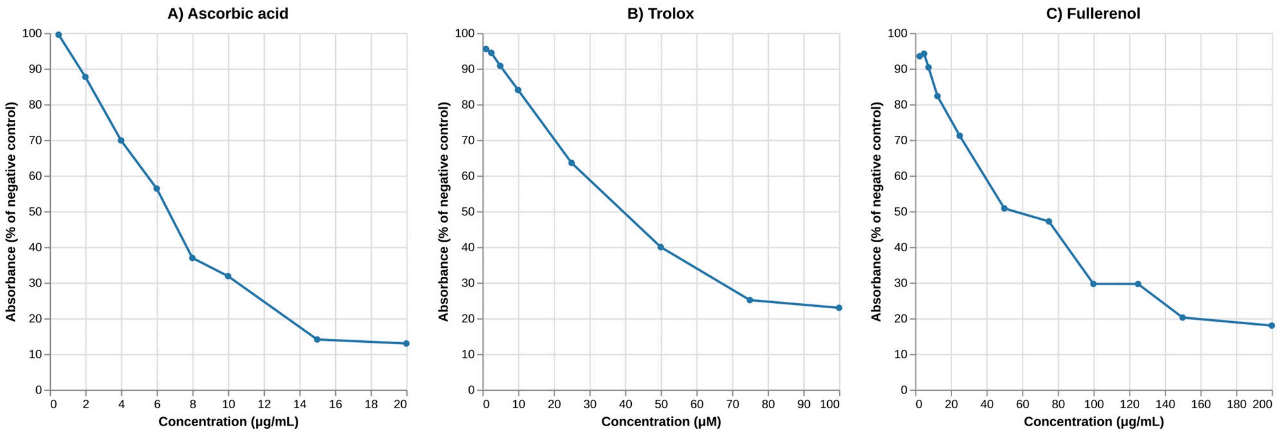

Figure S3. Change of absorbance in a DPPH-based assay of fullerenol antioxidant activity in comparison with Trolox and ascorbic acid (end-point measurement). N = 3. Mean values are shown.
