# Supplementary figures and images for: Effect of Fullerenol C60(OH)24 on Viability and Phagocytic Activity of Human Neutrophils
Source: Nanomaterials (Basel). 2026 Mar 27;16(7):405. doi: 10.3390/nano16070405 (PMC13075029; doi:10.3390/nano16070405)

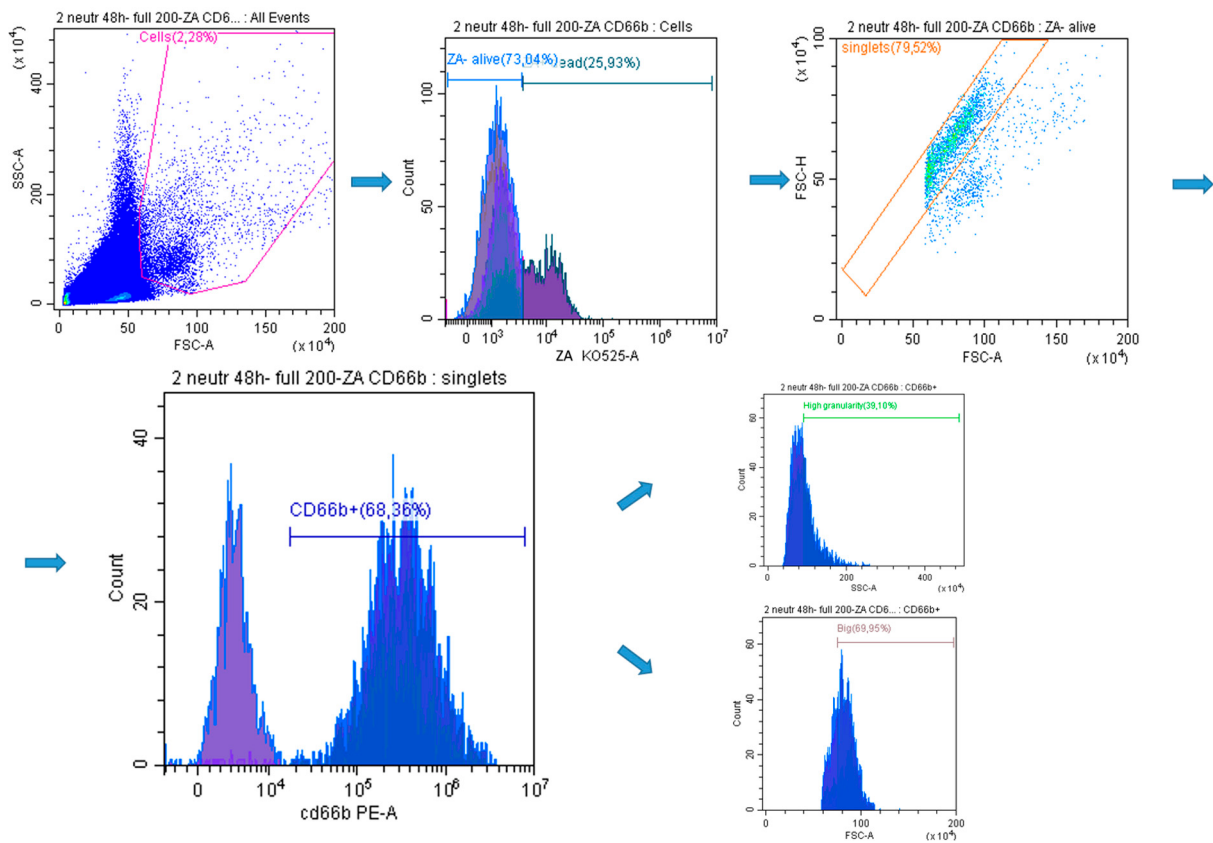

**Figure S1:** The gating strategy for the determination of highly granular and large neutrophils

Supplement: Supplementary file 1 [file nanomaterials-16-00405-s001.zip › Figure S1.pdf]
